# Limitations of Global Surveillance for *Neisseria gonorrhoeae* Antimicrobial Resistance

**DOI:** 10.3201/eid3206.260378

**Published:** 2026-06

**Authors:** Sebastiaan J. van Hal, Helen Fifer, Monica M. Lahra

**Affiliations:** Royal Prince Alfred Hospital, Sydney, New South Wales, Australia (S.J. van Hal); University of Sydney, Sydney (S.J. van Hal); United Kingdom Health Security Agency, London, UK (H. Fifer); Prince of Wales Hospital, Randwick, New South Wales, Australia (M.M. Lahra); University of New South Wales, Sydney (M.M. Lahra)

**Keywords:** Neisseria gonorrhoeae, antimicrobial resistance, bacteria, bacterial infections, sexually transmitted infections, Australia

## Abstract

*Neisseria gonorrhoeae* bacteria cause ≈82 million infections annually worldwide. As antimicrobial resistance (AMR) accelerates, the actual prevalence of antimicrobial-resistant infections remains obscured because of fragmented, heterogeneous, and often absent surveillance systems. The efficacy of ceftriaxone, widely used as first-line therapy, is increasingly threatened by the expansion of strains harboring the mosaic *penA* 60.001 allele, first documented in 2015 and now recognized globally as the dominant determinant of AMR. Our systematic search of the published literature identified 212 such isolates; nearly half were detected after 2022 in England and Australia, and epidemiologic data frequently linked acquisition of infection to the Asia-Pacific region. The substantially higher case numbers reported in those countries reflect the strength of their gonococcal AMR surveillance systems and more timely public data sharing. Our findings suggest that underreporting of the actual prevalence of ceftriaxone resistance is likely and that the opportunity for action is limited.

*Neisseria gonorrhoeae* bacteria are a priority pathogen for the World Health Organization (WHO) and cause an estimated 82 million new infections annually worldwide. Optimal disease control strategies remain unclear in light of increasing reports of antimicrobial resistance (AMR) and very limited global surveillance. A systematic review of surveillance systems for monitoring gonococcal AMR from 2022 found no evidence of a current surveillance system for gonorrhea in 148 countries, and only 6 countries (Australia, England, Wales, Scotland, Canada, and New Zealand) had gonococcal surveillance systems that were comprehensive and national. Those systems include all culture-based diagnoses of antimicrobial-susceptible gonorrhea infections and cover >50% of jurisdictions in their respective countries (*1*).

Historically, *N. gonorrhoeae* bacteria have developed resistance to every first-line therapeutic agent (*2*), including ceftriaxone, the current first-line treatment; newer agents such as gepotidacin and zoliflodacin show promise as alternatives. However, the longevity of the newer agents remains uncertain, particularly in settings where circulating *N. gonorrhoeae* isolates harbor the *parC* D86N mutation, 1 of 2 observed mutations required to develop resistance to the newer agents (*3*). In 2015, the emergence and subsequent spread of the ceftriaxone-resistant *N. gonorrhoeae* FC428 clone (i.e., strains harboring the ceftriaxone-resistance determinant) heralded the beginning of an era of increasing uncertainty regarding future antimicrobial drug management strategies (*4*,*5*). More than a decade later, the mosaic *penA* allele 60.001 persists as the prevailing determinant of ceftriaxone resistance (*6*).

We searched the published literature in June 2025 to identify reports of ceftriaxone-resistant *N. gonorrhoeae* bacteria harboring the *penA* 60.001 allele with linked genomic sequence data, to gain a contemporary understanding of AMR. We limited the search to isolates carrying *penA* 60.001 because other resistance-associated alleles, including the emerging *penA* 237.001 allele, remain relatively rare (*7*), whereas *penA* 60.001 has been consistently detected through routine surveillance across diverse settings. Although ceftriaxone-resistant *N. gonorrhoeae* has been reported by Canada, Japan, China, and WHO programs, the corresponding MICs and genomic sequencing data were not available (*8*–*12*).

We collated a total of 440 ceftriaxone-resistant isolates and defined resistance as an MIC >0.25 mg/L, regardless of the original susceptibility testing method used. Of the 440 isolates, 296 (67.3%) harbored the *penA* 60.001 allele, and genomic data were available for 212 (48.2%) isolates. Fifty percent (106/212) of all cases of ceftriaxone-resistant *N. gonorrhoeae* infection had been reported since 2022; 48% (51/106) of those were detected in England (n = 19) and Australia (n = 32). The remaining cases were detected in the Asia-Pacific region (n = 49), other countries in Europe (n = 4), and North America (n = 2) (*7*,*13*–*18*).

We constructed a phylogenetic tree, as previously described (*6*), following a mapping approach that used reference strain FA1090 (GenBank accession NC_002964.2) and masked recombination by using Gubbins version 2.12 (*19*). The phylogenetic analysis confirmed that the *penA* 60.001 allele associated with ceftriaxone resistance is predominantly in the Asia-Pacific region and that most cases detected elsewhere were linked to contact or travel in that region (Figure). The analysis also indicated that ceftriaxone resistance continues to evolve and has only a minority of recent isolates still clustering within the original FC428 clone. This trend of resistance evolution within multiple different clonal backbones signals an expanding and growing problem that is unlikely to be contained, portending serious challenges for therapy.

**Figure:**
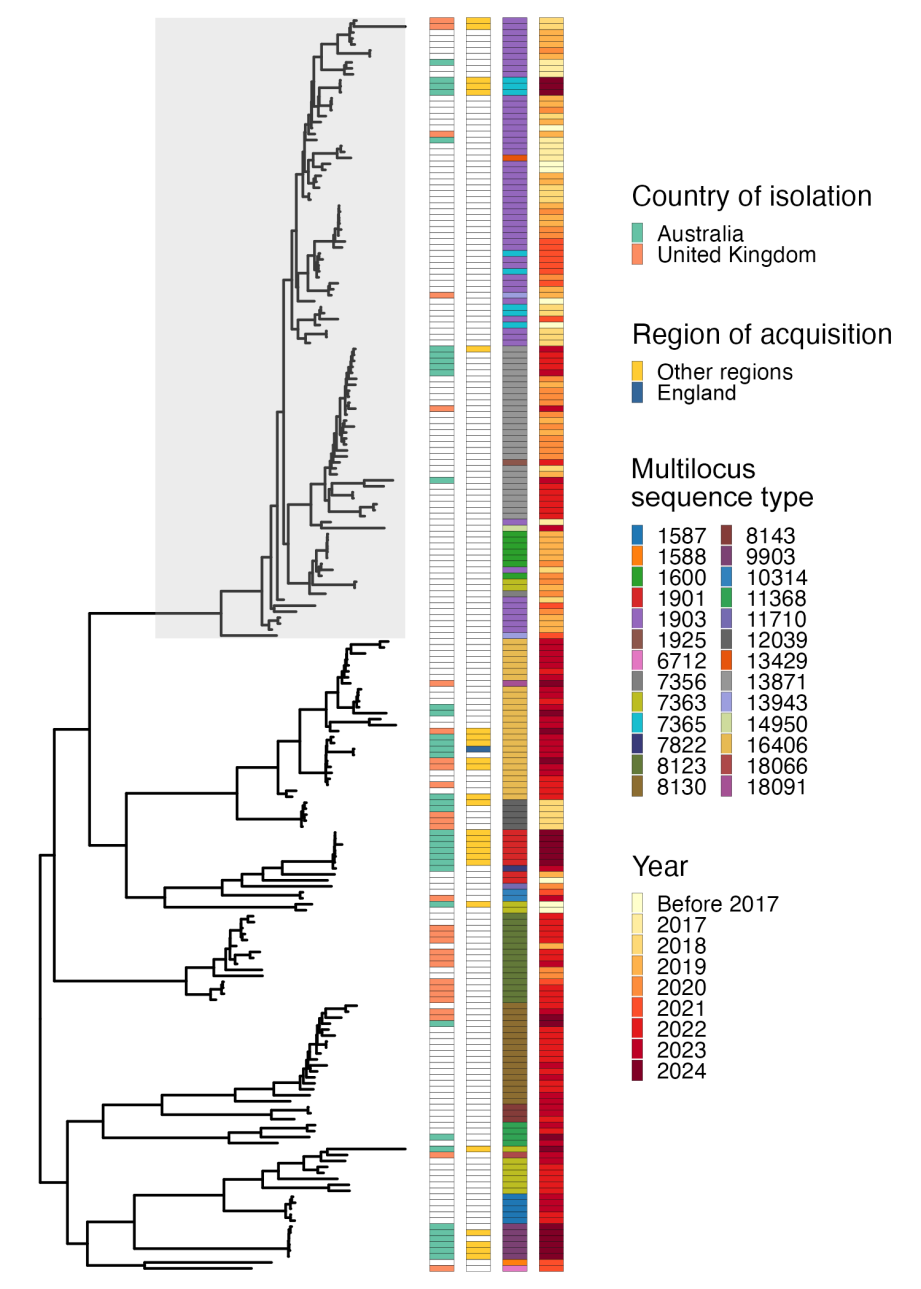
Maximum-likelihood phylogenetic tree of 212 ceftriaxone-resistant *Neisseria gonorrhoeae* isolates harboring *penA* 60.001 allele. Gray shading indicates original FC428 clone that emerged in 2015 in Japan and shows most new isolates clustering away from this clone. Columns at right shows isolates detected in England and Australia, reported regions where infections were acquired in Asia (countries include Cambodia, China, Indonesia, Japan, Thailand, Singapore, and Vietnam), multilocus sequence type, and year of isolation. Sequence types and isolate years demonstrate variation over time and genomic diversity.

Of note, most gonococcal infections are diagnosed by using molecular testing, and culture rates vary substantially across countries. Nevertheless, outside of the Asia-Pacific region, most reported cases of ceftriaxone-resistant *N. gonorrhoeae* infection are reported from England and Australia. One plausible explanation for the higher detection rates in those countries is the strength and coverage of their surveillance systems and the close integration of culture-based testing and antimicrobial susceptibility testing.

Gonococcal AMR in England and Wales is monitored through the Gonococcal Resistance to Antimicrobials Surveillance Programme, a sentinel surveillance system covering ≈2% of total yearly gonorrhea diagnoses (*20*). In addition, all cases of gonorrhea, regardless of infection site, have a sample taken for culture, and all ceftriaxone-resistant isolates in England are referred to the national Sexually Transmitted Infections Reference Laboratory for confirmation and follow-up (*21*). The number of ceftriaxone-resistant infections is reported on a quarterly basis. In Australia, culture is similarly recommended when feasible, and isolates are forwarded to the relevant jurisdictional *Neisseria* reference laboratories for antimicrobial susceptibility testing and follow-up. Australia’s jurisdictional *Neisseria* reference laboratories form a network, the National *Neisseria* Network, which is supported by the federal government and is responsible for the national surveillance system. National AMR surveillance data, derived from ≈24% of total annual cases, are publicly reported.

In contrast, even in countries with established surveillance systems, the low number of detected resistant isolates may primarily reflect underascertainment, resulting from the limited scope of culture-based surveillance, which typically tests or reports only a small fraction of cases through sentinel programs mainly targeting symptomatic men. Such restricted sampling might also explain why WHO-associated surveillance initiatives, including the Enhanced Gonococcal Antimicrobial Surveillance Programme in South-East Asia, have not detected higher rates of ceftriaxone resistance to date, except in Cambodia and Vietnam (*9*,*22*). Similarly, actual prevalence of antimicrobial-resistant *N. gonorrhoeae* infections in China remains unclear because accessible publications are largely regional and describe heterogeneous sampling strategies, limiting national-level interpretation (*8*,*23*). Alternatively, the routine public reporting of gonococcal AMR in China, especially when linked to genomic analyses, remains uncommon. As a result, antimicrobial-resistant cases may be identified locally but not reported widely.

Relying on the identification of treatment failure as an alternative signal of resistance is subject to many limitations. Only a small number of treatment failures have been reported globally, and those that have are almost exclusively in high-income countries, reflecting surveillance bias and underreporting. Detection of treatment failure is further complicated by inconsistent definitions of treatment failure, variability in treatment regimens, limited follow-up, and restricted access to culture-based diagnostics. As a result, AMR surveillance and treatment failure reporting probably underestimate the actual prevalence of ceftriaxone-resistant infections (*24*).

Therefore, for myriad reasons, the actual prevalence of gonococcal AMR is underestimated, which has prompted calls to expand AMR surveillance systems (*10*). However, such expansion is not always feasible, given social and political barriers, constraints in funding and laboratory capacity, and logistical challenges of scaling culture-based and antimicrobial susceptibility testing. In the absence of comprehensive global surveillance, data from England and Australia act as a canary in the coal mine, providing early warning of sentinel events. Furthermore, those datasets represent the best available evidence and should be used to model actual AMR prevalence and help guide international policy and intervention strategies.

The trajectory of *N. gonorrhoeae* AMR expansion is such that the window of opportunity for effective intervention is rapidly closing. Even with limited available global gonococcal AMR surveillance data, ceftriaxone resistance is high in some parts of the Asia-Pacific region; recent reports indicate resistance estimates of >20% (when applying an MIC >0.25 mg/L threshold) in some settings (*7*,*8*,*25*). The surveillance systems in England and Australia are signaling that ceftriaxone-resistant *N. gonorrhoeae* are transmitting globally and are originating from countries that report no ceftriaxone resistance. Furthermore, resistant isolates remain undetected in countries without comprehensive surveillance, meaning that the global gonococcal AMR prevalence exceeds current assumptions.
